# Hypertension: a cross-sectional study of the role of multimorbidity in blood pressure control

**DOI:** 10.1186/s12875-015-0313-y

**Published:** 2015-08-07

**Authors:** Chandra Sarkar, Hiten Dodhia, James Crompton, Peter Schofield, Patrick White, Christopher Millett, Mark Ashworth

**Affiliations:** King’s College London, Department of Primary Care and Public Health Sciences, Capital House, 42 Weston Street, London, SE1 3QD UK; Lambeth-Southwark Public Health Directorate, 160 Tooley Street, London, SE1 2QH UK; Imperial College London, Faculty of Medicine, School of Public Health, South Kensington Campus, London, SW7 2AZ UK

## Abstract

**Background:**

Hypertension is the most prevalent cardiovascular long-term condition in the UK and is associated with a high rate of multimorbidity (MM). Multimorbidity increases with age, ethnicity and social deprivation. Previous studies have yielded conflicting findings about the relationship between MM and blood pressure (BP) control. Our aim was to investigate the relationship between multimorbidity and systolic blood pressure (SBP) in patients with hypertension.

**Methods:**

A cross-sectional analysis of anonymised primary care data was performed for a total of 299,180 adult patients of whom 31,676 (10.6 %) had a diagnosis of hypertension. We compared mean SBP in patients with hypertension alone and those with one or more co-morbidities and analysed the effect of type of comorbidity on SBP. We constructed a regression model to identify the determinants of SBP control.

**Results:**

The strongest predictor of mean SBP was the number of comorbidities, β −0.13 (*p* < 0.05). Other predictors included Afro-Caribbean ethnicity, β 0.05 (*p* < 0.05), South Asian ethnicity, β −0.03 (*p* < 0.05), age, β 0.05 (*p* < 0.05), male gender, β 0.05 (*p* < 0.05) and number of hypotensive drugs β 0.06 (*p* < 0.05). SBP was lower by a mean of 2.03 mmHg (−2.22, −1.85) for each additional comorbidity and was lower in MM regardless of the type of morbidity.

**Conclusion:**

Hypertensive patients with MM had lower SBP than those with hypertension alone; the greater the number of MM, the lower the SBP. We found no evidence that BP control was related to BP targets, medication category or specific co-morbidity. Further research is needed to determine whether consultation rate, “white-coat hypertension” or medication adherence influence BP control in MM.

## Background

Multimorbidity (MM), the presence of more than one long-term condition (LTC), is common, increases with age, occurs 10–15 years earlier in deprived populations and is twice as prevalent in the non-white population [1–3]. The prevalence of MM is dependent on the definition [4]. A fairly narrow definition of MM has been adopted by the Quality and Outcomes Framework (QOF), a pay-for-performance incentive scheme introduced into the United Kingdom in 2004 with a focus on a series of clinical targets for the management of LTCs [5]. This scheme currently incorporates targets for 19 LTCs and in terms of blood pressure it is important to note that there are different BP targets for different conditions. The estimated prevalence of LTCs contained within the QOF in England is 16 % [5] and patients with a combination of these conditions account for 32 % of consultations in primary care [1]. Casting a wider net to include LTCs not within QOF, these proportions increase to 58 and 78 % respectively [1]. From the patient perspective, multimorbidity is associated with reduced functional status, poorer quality of life, increased mortality [5] and an increased use of inpatient and outpatient services [2].

Hypertension is the most common long-term condition in the UK, with 13.7 % of the population on the QOF hypertension register [6]. The true prevalence of hypertension is thought to be much higher with population studies such as the Health Survey for England estimating prevalence as high as 31 % in men and 27 % in women [7]. Hypertension is associated with a high level of multimorbidity and 78 % of hypertensive patients have at least one other LTC [2]. Hypertension is the single most important risk factor for the development of cardiovascular disease (CVD), with the risk of developing CVD doubling for every 20 mmHg increase in systolic blood pressure [8]. Hypertension is responsible for an estimated 51 % of stroke deaths and 45 % of IHD deaths worldwide [9].

Uncertainty exists about the relationship between blood pressure control and MM. Paulsen et al. [10] compared blood pressure control in over 37,000 Danish hypertensive patients and determined the proportion reaching their target blood pressure (BP), stratified according to multimorbidity. They demonstrated better BP control for some comorbidities (CVD, chronic obstructive pulmonary disease, osteoporosis, congestive heart failure and atrial fibrillation) and worse control for others (diabetes, diabetes with CVD, psychiatric disease, asthma and cancer). Although the sample size was large, findings may have been influenced by selection bias (48 % of the catchment population did not contribute data) and a relatively homogenous population in terms of ethnicity and deprivation. Two UK studies both concluded that BP control was improved in the presence of accompanying comorbidity but only cardiovascular comorbidities were included [3, 11].

Studies looking at health outcomes in other long-term conditions suggest worse outcomes compared to those with no LTCs. MM was shown to be a poor prognostic factor with regards to mortality and treatment complications in patients with cancer [12, 13], cardiovascular disease [14], diabetes [15] and common mental health problems [16]. Given this trend one would expect worse BP control with increased MM, contrary to the findings in the studies in hypertensive patients mentioned above.

Therefore, we aimed to determine the relationship between systolic blood pressure (SBP) which is associated with greater CVD risk compared to diastolic BP [17] in patients with hypertension and the number and type of multimorbidity. In particular we sought to identify whether combinations of multimorbidity such as cardiovascular, psychiatric or respiratory conditions influenced the relationship with BP control.

## Method

Data for this study were collected from Lambeth DataNet, a patient level primary care database containing information on patient demographics, QOF conditions, clinical measurements and medication. We used data from the year 2011/12 covering 299,180 patients over 18 years old registered at 51 of the 52 general practices in Lambeth (the remaining practice had an incompatible computer system). Lambeth is the ninth most deprived borough in London [18] and has the third highest proportion of Black African and Caribbean people in the UK at 25.9 % [19]. The patient characteristics for this population are shown in Table 1.

**Table 1:** Patient characteristics of the study population

Patient characteristics	Mean (SD)/proportion
Age (years)	63.88 (14.2)
Male	46.25 %
Female	53.75 %
IMD-2010	31.5 (8.7)
Number of comorbidities	0.83 (1.1)
White	43.9 %
Afro-Caribbean	33.7 %
South Asian	6.0 %

There were 31,793 persons on the QOF hypertension register, giving a recorded prevalence of 10.6 % in the adult population. This value is lower than estimates of national prevalence and reflects the relatively young population in Lambeth [20]. Data that were incorrectly coded and patients with no record of a BP reading or implausible BP readings (diastolic <30 or >200 mmHg; systolic <50 or >300 mmHg;), were excluded (*n* = 117). Inclusion of BP values was not influenced by the process of exception reporting, whereby GPs exclude some patients from QOF targets; our study included all BP data, regardless of whether the GP had exception reported the patient. Our final analysis was conducted on the remaining 31,676 cases with a recorded diagnosis of hypertension.

We selected twelve QOF conditions commonly included in studies of multimorbidity [2]. These included six cardiovascular comorbidities: ischaemic heart disease (IHD), heart failure, diabetes mellitus (DM), chronic kidney disease (CKD), stroke and atrial fibrillation (AF); three mental health comorbidites: depression, serious mental illness (SMI) and dementia; two respiratory comorbidities: chronic obstructive pulmonary disease (COPD) and asthma; and epilepsy. We then explored the pattern of BP control in hypertensive patients with and without multimorbidity and according to multimorbidity number, type and combination.

We calculated mean SBP for all patients with hypertension, using the last recorded SBP for each patient, and stratified the sample according to the presence of comorbidity, the number of comorbidities and their type. We used the analysis of covariance (ANCOVA) method to adjust the mean SBP values for age, ethnicity and gender. We also constructed linear regression models in order to identify the most important determinants of SBP in patients with hypertension using the ‘enter’ regression method. The following potential predictor variables were considered: age, gender, deprivation, ethnicity (White, Afro-Caribbean, South Asian and ‘Other’ on self reported ethnicity), medication (number of classes of hypotensive agents prescribed: ACE inhibitors, beta-blockers, calcium antagonists and diuretics) and number of comorbidities. Variables were only included in the model if they were found to be significant on univariate analysis, *p* < 0.1. Because of multiple testing, we only considered predictor variables in the regression model as significant if *p* <0.01. We conducted tests of colinearity and for the normality of residuals in order to ensure robustness of the model. Our analysis was conducted using SPSS version 20 [21].

## Results

In our sample of patients with hypertension, 16,140 (51 %) had one or more of the QOF co-morbidities. Patients with hypertension alone had a mean SBP of 139.4 mmHg (95 % CI; 139.2, 139.7) whereas those with any multimorbidity had a mean SBP of 136.3 mmHg (95 % CI; 136.1, 136.6).

**Fig. 1:**
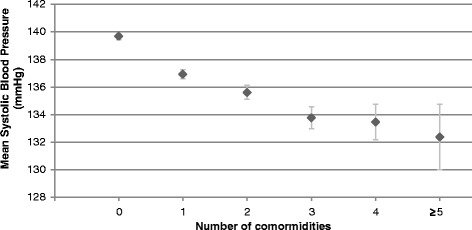
The relationship between number of comorbidities and mean systolic blood pressure in patients with hypertension. Error bars show the 95 % confidence interval of the mean

Patients with MM were further categorised by the number of comorbidities in addition to hypertension. The mean SBP of those with one comorbidity (*n* = 9626) was 137.1 mmHg (95 % CI 136.7, 137.4), for those with two comorbidities (*n* = 4028) was 136.0 mmHg (95 % CI 135.5, 136.5) and for those with three comorbidities was 134.3 mmHg (95 % CI 133.5, 135.2). Full results for patients with up to 5 or more comorbidities are displayed in Fig. 1.

**Fig. 2:**
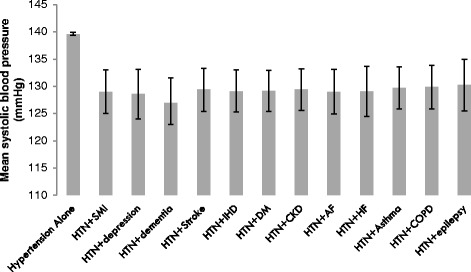
SBP values for patients with hypertension alone and with hypertension plus one comorbidity. (HTN = hypertension, SMI = serious mental illness, IHD = ischaemic heart disease, DM = diabetes, CKD = chronic kidney disease, AF = atrial fibrillation, COPD = chronic obstructive pulmonary disease). Error bars show the 95 % confidence interval of the mean (*n* = 9626)

**Fig. 3:**
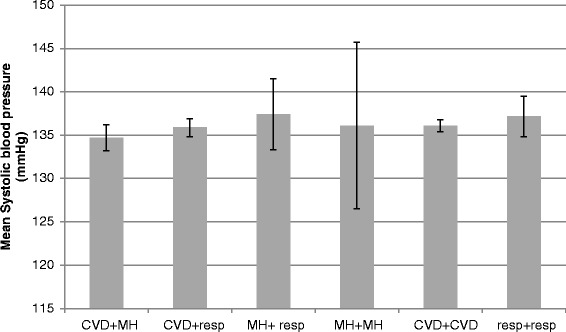
SBP values for patients with hypertension plus two comorbidities from the following groupings; CVD = cardiovascular disease (ischaemic heart disease, heart failure, diabetes, chronic kidney disease, stroke and atrial fibrillation) MH = mental health conditions (depression, dementia and serious mental illness), Resp = respiratory disease (chronic obstructive pulmonary disease and asthma). Error bars show the 95 % confidence interval of the mean (*n* = 4028)

The specific comorbidity made no difference to the mean SBP. There were no significant differences in blood pressure control between any of the individual comorbidities (Fig. 2) or between any of the comorbidity groupings in patients with hypertension plus two comorbidities (*n* = 4028) of cardiovascular, mental health and respiratory diseases (Fig. 3).

In our regression model, the strongest predictor of mean systolic BP was the number of comorbidities, B value =−2.03 (*p* < 0.05), beta =−0.13 (*p* < 0.05). Other significant predictors included the number of different hypotensive drug classes prescribed, ethnicity, gender and age (Table 2). The B values in Table 2 demonstrate the direct relationship between ethnicity and mean SBP; for example, the B value of 1.74 for Afro-Caribbean patients indicates that the mean SBP in the Afro-Caribbean sample was 1.74 mmHg higher than that of the White reference group. Social deprivation (IMD-2010) was not a significant determinant of systolic BP. There was no evidence of colinearity between the variables with a variance inflation factor (VIF) varying between 1.02 and 1.20 for the different variables. Analysis of the residuals showed a normal distribution with no relationship between the residuals (Durbin-Watson statistic 1.96).

**Table 2:** Predictors of SBP (unadjusted and adjusted) using multivariable regression

Predictors	B value, unstandardised (95 % confidence intervals)	Beta value, standardised	*p*-value
Number of comorbidities	−2.03 (−2.22, −1.85)	−0.13	<0.001
Number of classes of antihypertensive drugs	0.82 (0.66, 0.97)	0.06	<0.001
Age (years)	0.06 (0.04, 0.07)	0.05	<0.001
Gender (reference group male)	−1.63 (−2.00, −1.27)	−0.05	<0.001
South Asian ethnicity (reference group white)	−1.83 (−2.60, −1.07)	−0.03	<0.001
Afro Caribbean ethnicity (reference group white)	1.74 (1.35, 2.14)	0.05	<0.001
IMD score (IMD-2010)	0.008 (−0.01, 0.03)	0.004	0.043

## Discussion

Patients with hypertension with one or more comorbidities had a lower mean SBP than those with hypertension alone. The mean SBP was lower in patients with a greater number of comorbidities. Following adjustment for age, gender and ethnicity, the mean SBP was 2.03 mmHg lower for each additional morbidity. The improvement in BP control was independent of the type of comorbidity or comorbidity combination.

Our data provided further information on the factors with the potential to influence BP control. Firstly, we considered the effect of different QOF targets for SBP. We found no difference in mean SBP in patients with CKD or DM, both conditions for which the SBP QOF target is lower than for hypertension alone. Secondly, we found no evidence that the relationship between MM and SBP was mediated through the use of additional classes of hypotensive medication. Patients prescribed more classes of hypotensive agents had higher mean SBP. This is likely to be the result of reverse causality: that higher SBP resulted in more intensive hypotensive prescribing. Thirdly, we explored whether the overall relationship between MM and SBP might differ according to specific multimorbidity combinations. Morbidities which show a common pathophysiology, risk factor profile or management plan have been termed ‘concordant’; those that do not have been termed ‘discordant’ and in some studies these have been associated with poorer health outcomes [22]. There have been other studies, however where no association between ‘concordant’ and ‘discordant’ were found [23–25] and we found no difference in SBP attributable to concordant or discordant MM combinations with all comorbidities were associated with lower SBP compared to hypertension alone, regardless of the type of comorbidity (Figs. 2 and 3). The results demonstrate the achievement of primary care in controlling major risk factors of CVD in patients with multimorbidity and multiple riskfactors.

### Comparison with existing literature

Our results differed from the Danish study [10] in that we found a consistent reduction in SBP associated with comorbidity and that higher numbers of comorbidities were associated with a lower SBP. The difference in our results may be attributable to our more detailed analysis based on a continuous outcome variable (SBP) rather than dichotomised data - the Danish study only identified success or failure in achieving a BP target. Furthermore, by achieving high population coverage, our study had less potential for selection bias.

Other studies produced findings consistent with ours [2, 3, 11, 26] in terms of BP control in cardiovascular MM, but we were able to extend these findings by including respiratory and mental health comorbidities and epilepsy. In total, we analysed data for six non-cardiovascular MM and for each condition, we found a consistent association between additional co-morbidities and reductions in mean ‘‘SBP”.

### Strengths and limitations

Our study minimised selection bias though a large sample size covering 96 % of patients registered to practices in one inner London borough. This sample included a wide diversity in deprivation and ethnicity. We confined our analysis to QOF conditions which standardised the GP coding of these conditions. There are limitations in the interpretation of QOF data as it was not designed as a research tool and therefore the data are not externally validated [27] and inaccuracy in coding may also contribute to limitations of this data [28]. Our findings are likely to apply to the management of hypertension in other urban areas characterised by relatively high social deprivation and multi-ethnicity but it is less clear the extent to which they apply to more prosperous or predominantly mono-ethnic areas.

We considered other factors which we were unable to study because of the limitations of our dataset and which may have influenced the relationship between MM and SBP control. We were only able to use the last recorded SBP and not calculate an average over a period of time which may have given a better indication of overall SBP control. Patients with a greater number of comorbidities are likely to have higher consultation rates with health care professionals, be exposed to more opportunities for adjusting medication and discussing lifestyle measures, and more frequent BP recordings which in turn might reduce the ‘white coat’ effect on blood pressure readings and result in lower recorded SBP. Burstyn et al. suggested that patients who have their BP taken regularly are ‘trained’ and have a lower BP reading compared to those who are ‘untrained’ or not used to having their BP taken [29]. However, other studies have suggested that more frequent readings do not make a difference in ‘white-coat hypertension’ [30]. Alternatively, multimorbidity may be linked with increased adherence to hypotensive medication because of the perceived seriousness of multiple conditions [31]. Most patients with hypertension alone are asymptomatic, and this is likely to influence adherence [32]. Other studies, however, have found an association between multimorbidity and reduced adherence [16, 33]. Although we were able to record the category of hypotensive medication, we were not able to convert this to a standardised measure of daily dosage nor were we able to measure medication adherence. Furthermore, we were unable to access data on the consultation rates for patients with different morbidities.

## Conclusion

Our findings demonstrate that overall BP control is improved in multimorbidity patients seen in primary care. The findings are reassuring given the growing number of people with MM, but whether improved intermediate outcome measures in patients with MM translate into improved cardiovascular health outcomes needs to be explored. Following these findings which in some ways were counter-intuitive, we plan to extend out work to look at other intermediate outcomes such as HbA1c and cholesterol levels. National data on consultation rates have not been produced since 2008 [34] and in future work we plan to collect local data on consultation rates to explore this important confounder. The role of multimorbidity in the management of cardiovascular risk factors is complex but is an important component of primary care and needs to be reflected in clinical guidance, training and improvement initiatives [35, 36].

### Ethical approval

Ethical approval was given by the South East ResearchEthics Committee-07MRE01/26.

## Abbreviations

MM: Multimorbidity
BP: Blood pressure
SBP: Systolic blood pressure
LTC: Long term condition
CVD: Cardiovascular disease
QOF: Quality outcomes framework
IHD: Ischaemic heart disease
CKD: Chronic kidney disease
AF: Atrial fibrillation
SMI: Serious mental illness
COPD: Chronic obstructive pulmonary disease
DM: Diabetes mellitus
ANCOVA: Analysis of covariance

## References

[1] Salisbury C, Johnson L, Purdy S, Valderas JM, Montgomery AA (2011). Epidemiology and impact of multimorbidity in primary care: a retrospective cohort study. BJGP.

[2] Barnett K, Mercer SW, Norbury M, Watt G, Wyke S, Guthrie B (2012). Epidemiology of multimorbidity and implications for health care, research, and medical education: a cross-sectional study. Lancet.

[3] Mathur R, Hull SA, Badrick E, Robson J (2011). Cardiovascular multimorbidity: the effect of ethnicity on prevalence and risk factor management. BJGP.

[4] Fortin M, Stewart M, Poitras ME, Almirall J, Maddocks H (2012). A systematic review of prevalence studies on multimorbidity: toward a more uniform methodology. Ann Fam Med.

[5] Smith SM, O’Dowd T (2007). Chronic diseases: what happens when they come in multiples?. BJGP.

[6] Health and Social Care Information Centre. Disease Prevalence Quality and Outcomes Framework for April 2012-March2013, England. 2013 [cited 21/12/2013]. Available from: http://www.hscic.gov.uk/searchcatalogue?productid=12972&q=hypertension&sort=Relevance&size=10&page=1#top.

[7] Health and Social Care Information Centre. Health Survey for England - 2012, Trend tables 2013 [cited 04/06/2014]. Available from: http://www.hscic.gov.uk/catalogue/PUB13219.

[8] Schofield P, Baawuah F, Seed PT, Ashworth M (2012). Managing hypertension in general practice: a cross-sectional study of treatment and ethnicity. BJGP.

[9] World Health Organisation (2013). A global brief on hypertension. Silent killer, global public health crisis.

[10] Paulsen MS, Andersen M, Thomsen JL, Schroll H, Larsen PV, Lykkegaard J (2013). Multimorbidity and Blood Pressure Control in 37 651 Hypertensive Patients From Danish General Practice. Journal of the American Heart Association.

[11] Millett C, Gray J, Bottle A, Majeed A (2008). Ethnic disparities in blood pressure management in patients with hypertension after the introduction of pay for performance. Ann Fam Med.

[12] Lund L, Jacobsen J, Nørgaard M, McLaughlin JK, Blot WJ, Borre M (2009). The prognostic impact of comorbidities on renal cancer, 1995 to 2006: a Danish population based study. J Urol.

[13] Piccirillo JF, Vlahiotis A (2006). Comorbidity in patients with cancer of the head and neck: prevalence and impact on treatment and prognosis. Curr Oncol Reports.

[14] Glynn LG, Buckley B, Reddan D, Newell J, Hinde J, Dinneen SF (2008). Multimorbidity and risk among patients with established cardiovascular disease: a cohort study. Br J Gen Pract.

[15] Austin RP (2006). Polypharmacy as a risk factor in the treatment of type 2 diabetes. Diabetes Spectrum.

[16] Bautista LE, Vera-Cala LM, Colombo C, Smith P (2012). Symptoms of depression and anxiety and adherence to antihypertensive medication. Am J Hypertens.

[17] Stamler J, Stamler R, Neaton JD (1993). Blood pressure, systolic and diastolic, and cardiovascular risks: US population data. Arch Intern Med.

[18] Department for communities and local government. English Indicies of deprivation 2010. England 2011. [cited 03/03/2014]. Available from: https://www.gov.uk/government/uploads/system/uploads/attachment_data/file/6871/1871208.pdf.

[19] Office for National Statistics. 2011 Census: KS201UK Ethnic group, local authorities in the United Kingdom. 2011. Available from: http://www.ons.gov.uk/ons/datasets-and-tables/index.html?pageSize=50&sortBy=none&sortDirection=none&newquery=ethnic+lambeth.

[20] Lambeth Council. 2012. State of the Borough Report. London: Lambeth Council.

[21] IBM Corp. SPSS Statistics for Windows, Version 20.0. Armonk, NY: IBM Corp. Released 2011

[22] Piette JD, Kerr EA (2006). The Impact of Comorbid Chronic Conditions on Diabetes Care. Diabetes Care.

[23] Lagu T, Weiner MG, Hollenbeak CS, Eachus S, Roberts CS, Schwartz JS (2008). The impact of concordant and discordant conditions on the quality of care for hyperlipidemia. J Gen Intern Med.

[24] Magnan EM, Palta M, Johnson HM, Bartels CM, Schumacher JR, Smith MA (2014). The impact of a patient’s concordant and discordant chronic conditions on diabetes care quality measures. J Diabetes Complications.

[25] Kerr EA, Heisler M, Krein SL, Kabeto M, Langa KM, Weir D (2007). Beyond comorbidity counts: how do comorbidity type and severity influence diabetes patients’ treatment priorities and self-management?. J Gen Intern Med.

[26] Mathur R, Hull SA, Boomla K, Robson J (2012). Ethnic differences in primary care management of diabetes and cardiovascular disease in people with serious mental illness. Brit J Gen Pract.

[27] Ashworth M, Medina J, Morgan M. Effect of social deprivation on blood pressure monitoring and control in England: a survey of data from the quality and outcomes framework 2008 2008-10-29 01:00:42. 10.1136/bmj.a2030 PMC2590907 18957697

[28] Khan NF, Harrison SE, Rose PW. Validity of diagnostic coding within the General Practice Research Database: a systematic review 2010 2010-03-01 00:00:00. e128–e36. 10.3399/bjgp10X483562 PMC2828861 20202356

[29] Burstyn P, O’Donovan B, Charlton I (1981). Blood pressure variability: the effects of repeated measurement. Postgrad Med J.

[30] Pickering TG, James GD, Boddie C, Harshfield GA, Blank S, Laragh JH (1988). HOw common is white coat hypertension?. JAMA.

[31] DiMatteo MR, Haskard KB, Williams SL (2007). Health beliefs, disease severity, and patient adherence: a meta-analysis. Med Care.

[32] Marshall IJ, Wolfe CD, McKevitt C (2012). Lay perspectives on hypertension and drug adherence: systematic review of qualitative research. BMJ.

[33] Mercer S, Salisbury C, Fortin M. 2014. ABC of Multimorbidity: John Wiley & Sons, London.

[34] Health and Social Care Information Centre. Trends in Consultation Rates in General Practice - 1995–2009. 2009 [cited 20/06/2015]. Available from: http://www.hscic.gov.uk/pubs/gpcons95-09.

[35] Guthrie B, Payne K, Alderson P, McMurdo MET, Mercer SW. Adapting clinical guidelines to take account of multimorbidity. BMJ. 2012 2012-10-04 12:03:03;345. 10.1136/bmj.e6341 23036829

[36] Payne RA, Abel GA, Guthrie B, Mercer SW (2013). The effect of physical multimorbidity, mental health conditions and socioeconomic deprivation on unplanned admissions to hospital: a retrospective cohort study. Can Med Assoc J.

